# Supplementary material for: Effect of CXCL17 on Subcutaneous Preadipocytes Proliferation in Goats
Source: Animals (Basel). 2023 May 25;13(11):1757. doi: 10.3390/ani13111757 (PMC10252012; doi:10.3390/ani13111757)
Supplement: Supplementary file 1 [file animals-13-01757-s001.zip › animals-2347595-supplementary.pdf]

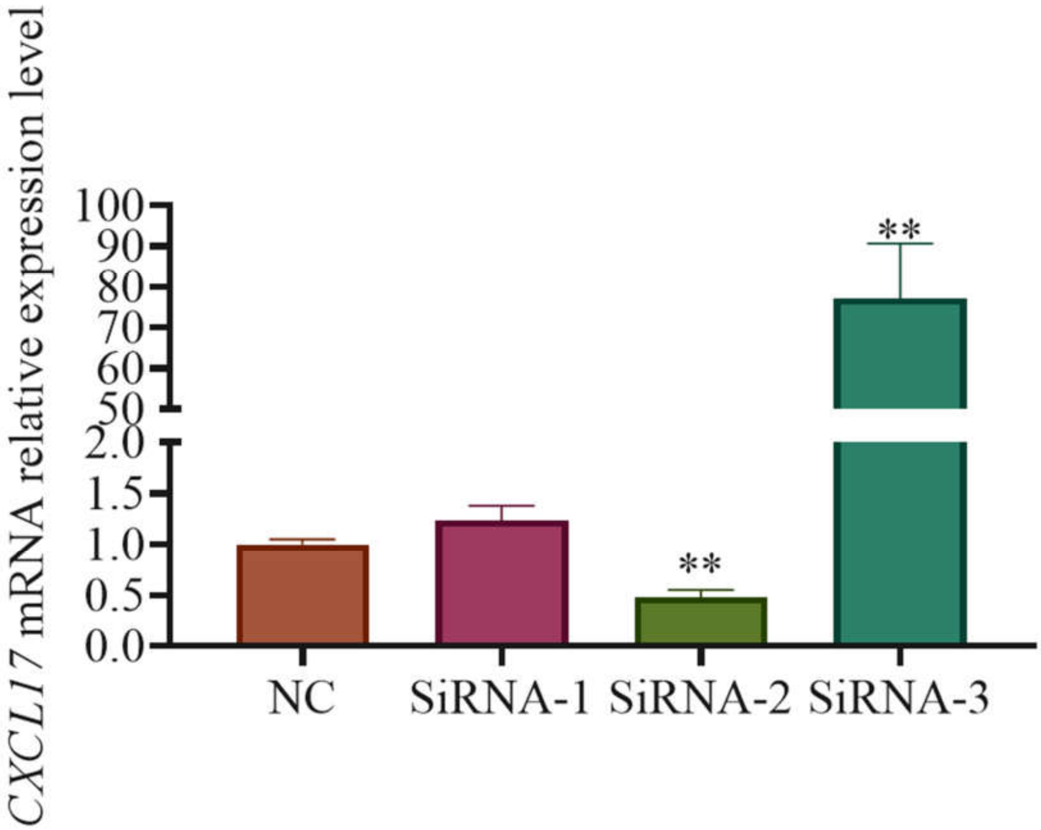

Figure S1. The knockdown efficiency detection of *CXCL17* at mRNA, SiRNA-1, SiRNA-2 and SiRNA-3 are synthesized by Shanghai GenePharma Co.,Ltd. (Shanghai, China), **: p <0.01.
